# Serum MicroRNA Expression Patterns in Subjects After the 5-km Exercise Are Strongly Associated With Cardiovascular Adaptation

**DOI:** 10.3389/fphys.2021.755656

**Published:** 2021-11-29

**Authors:** Dandan Li, Pingping Wang, Wenyan Wei, Cheng Wang, Yong Zhong, Lei Lv, Junjun Wang

**Affiliations:** ^1^Department of Clinical Laboratory, Jinling Hospital, Nanjing University School of Medicine, Nanjing, China; ^2^Department of Health Medicine, Jinling Hospital, Nanjing University School of Medicine, Nanjing, China; ^3^Department of Geriatric Cardiology, Jinling Hospital, Nanjing University School of Medicine, Nanjing, China

**Keywords:** 5-km run test, exercise, microRNAs, cardiovascular disease, adaptation

## Abstract

Circulating microRNAs (miRNAs) have been reported dysregulated during exercise. However, the changes of specific serum miRNAs during the 5-km run test with intensity of 51–52% maximum oxygen uptake (V̇O_2_max) and their association with traditional cardiovascular-related indicators remain well-characterized. Levels of miR-1, miR-21, miR-146a, miR-155, miR-181, and miR-210 were detected in 120 young subjects before and after the exercise training by quantitative reverse-transcription PCR (RT-qPCR). Besides, the levels of cardiac troponin I (cTNI), myoglobin (Myo), creatine kinase (CK), creatine kinase-MB (CK-MB), aspartate aminotransferase (AST), lactate dehydrogenase (LDH), ischemia-modified albumin (IMA), interleukin-6 (IL-6), and C-reactive protein (CRP) were measured and the correlation between levels of serum miRNAs and biochemical parameters was also analyzed. Compared with resting state, the serum levels of miR-1, miR-146a, miR-155, miR-181, and miR-210 were significantly increased after exercise training. Serum levels of miR-146a, miR-155, and miR-210 after exercise training were positively correlated with Myo, CK-MB, and LDH, respectively, while miR-1, miR-146a, miR-181, and miR-155 were positively correlated with the levels of IL-6. Additionally, all the five miRNAs were negatively correlated with IMA levels. The multivariate logistic regression analysis showed that high levels of miR-146a, AST, LDH, and IL-6 in serum were risk factors, while low IMA contents were a protective factor for cardiovascular adaptation during exercise. In conclusion, the dynamic changes of miRNAs under the condition of the 5-km continuous running contribute to the adaptive regulation of the cardiovascular function of the body.

## Introduction

The 5-km run test is the basic training item and the main content of endurance training for subjects. Studies have shown that moderate physical exercise can lead to adaptive changes in the cardiovascular system of the body, such as myocardial angiogenesis, increased coronary blood flow, and reduced inflammatory response in the body (Yu et al., 2017; Min et al., 2019; Schuttler et al., 2019). Although many studies have elucidated the protective effect of regular aerobic exercise on myocardium and the promotion of heart health, the underlying molecular mechanism causing this adaptive change is unclear (Schuler et al., 2013). Therefore, understanding the cardiovascular adaptation of subjects after training is a key issue that should be studied.

In recent years, microRNAs (miRNAs) have been found to be dynamically dysregulated in response to physiological and pathological processes (Wang et al., 2018). Numerous studies demonstrated that miRNAs are novel indicators for cardiovascular disease (CVD) risk prediction, condition monitoring, and prognosis, which can regulate heart development and physiological function (Ultimo et al., 2018). Meanwhile, miRNAs have a higher diagnostic value for CVD when compared with the established gold standard (de Gonzalo-Calvo et al., 2017). Recently, it reports that exercise alters the miRNAs profile and these altered miRNAs have been observed to participate in metabolic pathways related to myocardial function, metabolism, and inflammatory response (Schuler et al., 2013). Till date, dynamic regulation of miRNAs during exercise in healthy subjects and athletes has been analyzed and the response of circulating miRNAs to exercise in patients with CVD is determined (Xu et al., 2016; Li et al., 2018; Zhou et al., 2020). However, no robust correlation was identified between changes of these miRNAs and myocardial marker, inflammation indicator, or ischemia marker, indicating that further studies are still required to identify the potential use of the circulating miRNAs of exercise.

Exercise induced alterations of miRNAs in skeletal tissue compartments including cardiac cell, vascular endothelium, and inflammatory cells. However, the expression profiles of miRNA were different in various types and intensities of exercise (Ultimo et al., 2018). In addition, the response of circulating miRNAs to the 5-km run test in subjects remains undetermined. In this study, we screened six representative miRNAs associated with the occurrence, development, and regulation of cardiovascular diseases through literature review, which enriched in muscle (miR-1) (Jiang et al., 2020), vascular endothelium (miR-155) (Kolahi et al., 2018), associated with inflammation (miR-21, miR-146a, and miR-181) (Li et al., 2007; Gangwar et al., 2018; Kolahi et al., 2018; Song et al., 2019), and ischemia hypoxia adaptation (miR-210) (Fasanaro et al., 2008), with the purpose to investigate how specific circulating miRNAs with well-established roles in the adaptive process is linked to the 5-km run test in subjects.

In this study, we quantify the above expression of candidate miRNAs during long-term aerobic exercise and further explored their correlations with traditional cardiovascular disease indicators. The aim of this study to look for key indicators that affect the cardiovascular adaptive capacity of the body before and after aerobic exercise, which is expected to provide novel insight for scientific and reasonable training in the future.

## Materials and Methods

### Study Subjects

A total of 120 young male subjects randomly selected from a certain army who run the 5-km test in 24 min in Nanjing. All the subjects had been conducted regular training for 1 month. Subjects with coronary heart disease, hypertension, diabetes, stroke, and other diseases were excluded from this study. The basic information was shown in Table 1. Additionally, the average duration of exercise training was 21 min and the average heart rate was 170 beats/min. Meanwhile, the average exercise intensity was 51–52% VO_2_ max and the maximum exercise intensity was 70% VO_2_ max. This study was approved by the Medical Ethics Committee (2018NZGKJ-096) of the Jinling Hospital. A written informed consent was obtained from all the subjects.

**TABLE 1:** Characteristics of study subjects.

Parameters	(*n* = 120)
Age (years)	19.00 (18.25, 20.00)
Height (cm)	170.00 ± 5.72
Weight (kg)	60.8 (57.0, 64.0)
BMI (kg/m^2^)	20.85 (19.83, 22.13)
Systolic blood pressure (mmHg)	115.10 ± 9.20
Diastolic blood pressure (mmHg)	63.16 ± 8.77

### Sample Collection

A total of 3.5 ml of elbow vein blood samples were collected from 120 subjects in 30 min before and after training, respectively. After the blood samples were collected, the serum was centrifuged at 3,000 rpm for 5 min and separated at room temperature rapidly and the biochemical parameters were tested on the same day or the serum samples were stored at −80^°^C until analyzed.

### Serum miRNAs Detection

The serum total RNA was extracted by the one-step phenol/chloroform purification according to our previously described protocol (Yan et al., 2019). Reverse transcription was performed using avian myeloblastosis virus reverse transcriptase (Takara, Otsu, Japan) and a stem-loop RT primer (Applied Biosystems, Foster City, CA, United States). Quantitative PCR was performed as the following: 95^°^C for 5 min, 40 cycles of 95^°^C for 15 s, and 60^°^C for 1 min in a total of 20 μl reaction. All the reactions were conducted in triplicate. Relative levels of targeted miRNAs were normalized to exogenous control MiR2911 (5′-GGCCGGGGGACGGGCUGGGA-3′) and were calculated using the comparative Ct method (2^–ΔΔCt^), in which the ΔCt was calculated by subtracting the Ct values of MiR2911 from the Ct values of the target miRNAs.

### Measurement of the Clinical Biochemical Parameters

The serum levels of cardiac troponin I (cTNI) and myoglobin (Myo) were determined with chemiluminescence by the Tosoh Bioscience AIA-2000 Automated Immunoassay Analyzer using commercial reagents (Tosoh Corporation, Tokyo, Japan). Creatine kinase (CK), creatine isoenzyme (CK-MB), aspartate aminotransferase (AST), and lactate dehydrogenase (LDH) were measured on a 7600 Hitachi Automatic Analyzer with commercial reagents (Hitachi High-Technologies Corporation, Tokyo, Japan). Interleukin-6 (IL-6) was studied with Roche diagnostic kits in the Cobas e-411 Roche Auto Analyzer. Serum IMA was analyzed using principle of albumin-cobalt binding defined by Nazik et al. (Nazik et al., 2017) on the Mindray BS-2000M Auto Analyzer (Mindray, Shenzhen, China) and C-reactive protein (CRP) was measured using the Mindray BC-5390 Auto Hematology Analyzer (Mindray, Shenzhen, China).

### Statistical Analysis

Data analysis was performed by the Statistical Package for the Social Sciences (SPSS) version 21.0 software. All the data were tested for normality and variance homogeneity. The data consistent with the normal distribution were expressed as mean ± SD and comparison between the two groups using independent samples *t*-test analysis. The Fisher’s least significant difference (LSD) test was used for homogeneity of variance and the Tamhane’s test was used for non-homogeneity of variance. Data with non-normal distribution were expressed as median (interquartile spacing) [M (P25, P75)] and the non-parametric Mann–Whitney *U* test was used for comparison between the two groups. The Spearman’s rank correlation analysis was used for correlation analysis among variables and the logistic regression analysis was used for CVD risk factors. Bilateral test was used and *p* < 0.05 was considered as statistically significant.

## Results

### Analysis of Levels of Serum miRNAs

**FIGURE 1:**
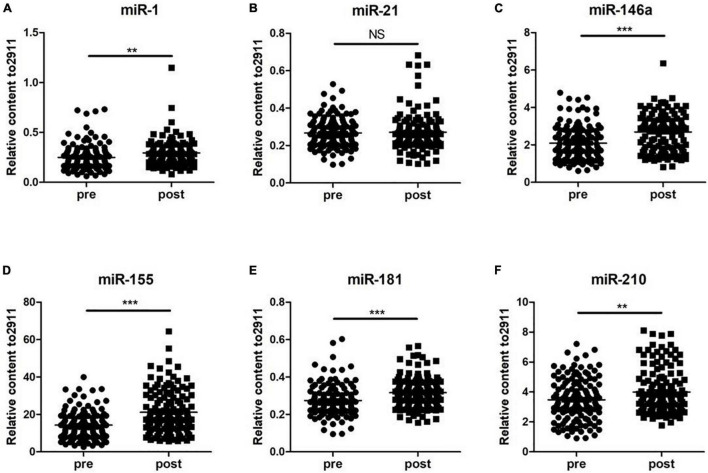
Changes in circulating microRNAs in response to the 5-km run test in 120 young soldiers. The expression levels were evaluated before (pre) and immediately after (post) the 5-km run test. Each point represents the mean of triplicate samples. **(A)** miR-1; **(B)** miR-21; **(C)** miR-146a; **(D)** miR-155; **(E)** miR-181; and **(F)** miR-210. The *p*-value was derived from the non-parametric Mann–Whitney *U* test between the pro- and postgroups. **p < 0.01; ***p < 0.001.

To determine whether the selected miRNAs were altered during exercise, two groups, including 120 subjects before and after the 5-km run test, were analyzed. The results showed that the levels of miR-1, miR-146a, miR-155, miR-181, and miR-210 after training were significantly higher than those before the 5-km run test (*p* < 0.05). However, the levels of miR-210 in serum of 120 subjects were not found to be significantly changed during exercise (Figure 1). These results suggested that the altered miRNAs may be important indicators of training adaptation.

### Detection of Clinical Biochemical Parameters

**FIGURE 2:**
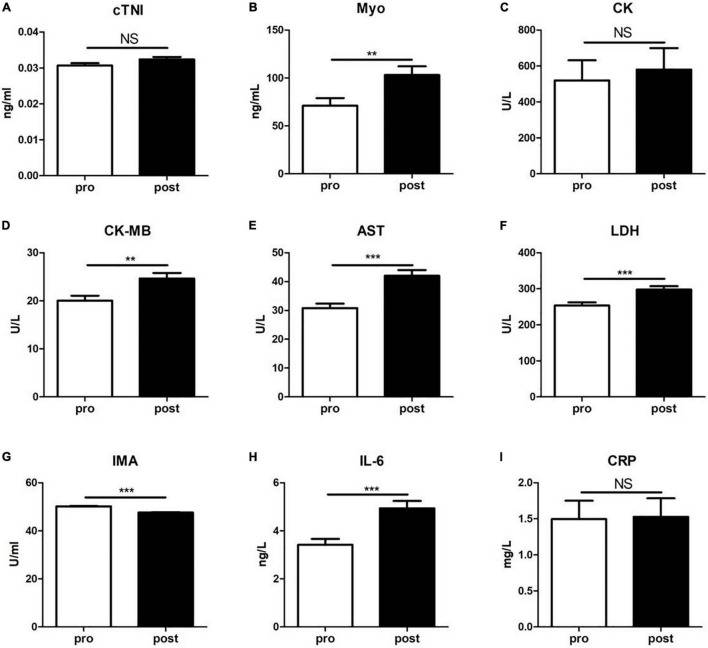
The levels of a panel of cardiovascular diseases-associated clinical biochemical parameters were evaluated before (pre) and immediately after (post) the 5-km run test. **(A)** cardiac troponin I (cTNI); **(B)** myoglobin (Myo); **(C)** creatine kinase (CK); **(D)** creatine isoenzyme (CK-MB); **(E)** aspartate aminotransferase (AST); **(F)** lactate dehydrogenase (LDH); **(G)** ischemia-modified albumin (IMA); **(H)** interleukin-6 (IL-6); and **(I)** C-reactive protein (CRP). Data were analyzed using the unpaired *t*-test. The results are presented as the mean ± SEM, **p < 0.01; ***p < 0.001.

We next examined the clinical biochemical parameters that are associated with CVD and compared their changes before and after training. As shown in Figure 2, the serum levels of Myo, CK-MB, AST, LDH, and IL-6 in subjects after exercise were significantly increased than those before exercise, while the serum levels of IMA were decreased. However, there was no significant difference of cTNI, CK, and CRP levels between the two groups.

### Associations Between miRNAs and Clinical Biochemical Parameters

To further evaluate the relationship among the altered miRNAs and cardiovascular-associated parameters, the Spearman’s rank correlation analyses were performed in the two groups. The miR-1 levels were found positively correlated with IL-6 (*r* = 0.178, *p* = 0.006), while negatively correlated with IMA (*r* = −0.213, *p* < 0.001). The miR-146a levels were positively correlated with Myo (*r* = 0.181, *p* = 0.005) and IL-6 (*r* = 0.244, *p* = 0.000), while negatively correlated with IMA (*r* = −0.230, *p* = 0.000). The miR-155 levels were positively correlated with LDH (*r* = 0.143, *p* = 0.027) and IL-6 (*r* = 0.179, *p* = 0.006), while negatively correlated with IMA (*r* = −0.248, *p* = 0.000). The miR-181 levels were positively correlated with IL-6 (*r* = 0.195, *p* = 0.002), while negatively correlated with IMA (*r* = −0.240, *p* = 0.000). Meanwhile, the miR-210 levels were positively correlated with CK-MB (*r* = 0.149, *p* = 0.021) and negatively correlated with IMA (*r* = −0.147, *p* = 0.023), respectively (Table 2).

**TABLE 2:** The Spearman’s rank correlation coefficients between altered microRNAs and cardiovascular diseases-associated clinical biochemical parameters in 120 young soldiers.

Variables	Myo	CK-MB	AST	LDH	IL-6	IMA
miR-1	r = 0.105 *p* = 0.105	r = 0.115 *p* = 0.075	r = 0.087 *p* = 0.180	r = −0.084 *p* = 0.195	r = 0.178 *p* = 0.006	r = −0.213 *p <* 0.001
miR-146a	r = 0.181 *p* = 0.005	r = 0.123 *p* = 0.057	r = 0.028 *p* = 0.663	r = 0.089 *p* = 0.173	r = 0.244 *p* = 0.000	r = −0.230 *p* = 0.000
miR-155	r = 0.016 *p* = 0.809	r = −0.003 *p* = 0.966	r = −0.058 *p* = 0.370	r = 0.143 *p* = 0.027	r = 0.179 *p* = 0.006	r = −0.248 *p* = 0.000
miR-181	r = 0.082 *p* = 0.206	r = 0.125 *p* = 0.053	r = 0.049 *p* = 0.451	r = 0.123 *p* = 0.058	r = 0.195 *p* = 0.002	r = −0.240 *p* = 0.000
miR-210	r = 0.109 *p* = 0.092	r = 0.149 *p* = 0.021	r = 0.058 *p* = 0.372	r = 0.030 *p* = 0.640	r = 0.016 *p* = 0.809	r = −0.147 *p* = 0.023

*Myo, myoglobin; CK-MB, creatine isoenzyme; AST, aspartate aminotransferase; LDH, lactate dehydrogenase; IL-6, interleukin-6; IMA, ischemia-modified albumin.*

### Logistic Regression Analysis of Factors Affecting Cardiovascular Adaptation During Exercise

Subsequently, the univariate and multivariate logistic regression analyses were performed to identify altered miRNAs and clinical biochemical parameters associated with the cardiovascular system during training in 120 subjects. The binary variables of the two groups before and after training were taken as dependent variables and serum miRNAs, Myo, CK-MB, AST, LDH, IL-6, and IMA as independent variables. In the univariate logistic regression analysis, serum miR-1, miR-146a, miR-155, miR-181, miR-210, Myo, CK-MB, AST, LDH, IL-6, and IMA levels were included, respectively. The results showed that all the included indicators, except miR-210, were associated with the cardiovascular adaptation process after exercise. Further, the multivariate logistic regression analysis showed that after adjusting for the other observed indicators, high miR-146a, AST, LDH, and IL-6 levels were risk factors, while low IMA was a protective factor for cardiovascular adaptation during exercise (Table 3).

**TABLE 3:** The univariate and multivariate logistic regression analysis of risk factors for cardiovascular diseases in 120 young soldiers during the 5-km run test.

Variables	Univariate analysis		Multivariate analysis	
	OR (95%CI)	*P*	OR (95%CI)	*P*
miR-1	26.038 (2.996, 226.292)	0.003		
miR-146a	1.517 (1.197, 1.922)	0.001	1.919 (1.348, 2.733)	0.000
miR-155	1.036 (1.015, 1.059)	0.001		
miR-181	37.380 (2.630, 531.218)	0.007		
Myo	1.014 (1.005, 1.023)	0.002		
CK-MB	1.093 (1.036, 1.152)	0.001		
AST	1.060 (1.033, 1.088)	0.000	1.067 (1.031, 1.104)	0.000
LDH	1.012 (1.006, 1.018)	0.000	0.993 (0.986, 1.000)	0.048
IL-6	1.204 (1.086, 1.336)	0.000	1.383 (1.190, 1.607)	0.000
IMA	0.528 (0.444, 0.627)	0.000	0.454 (0.359, 0.575)	0.000

*Myo, myoglobin; CK-MB, creatine isoenzyme; AST, aspartate aminotransferase; LDH, lactate dehydrogenase; IL-6, interleukin-6; IMA, ischemia-modified albumin.*

## Discussion

In this study, we analyzed the profile of a panel of circulating miRNAs that were proposed involving in cardiovascular physiology and the pathogenesis of CVD in response to exercise, together with a series of clinical biochemical parameters associated with cardiovascular disease, in healthy young male subjects. Our results showed that specific miRNAs are associated with clinical biochemical parameters and may participate in the regulation of cardiovascular adaptive capacity during the 5-km run test.

In recent years, scientists have paid much attention to the role of circulating miRNAs during the process of exercise adaption in an attempt to serve as a potential marker to guide the treatment of diseases and the training effect of athletes (Clauss et al., 2016; de Gonzalo-Calvo et al., 2017; Mayr et al., 2019). However, there was little known about the circulating miRNAs expression profiles after the 5-km run test. In this study, we analyzed and compared the circulating serum levels of miR-1, miR-21, miR-146a, miR-155, miR-181, and miR-210 of the subjects before and after exercise training. As a result, five miRNAs including miR-1, miR-146a, miR-155, miR-181, and miR-210 showed a significant increase after the 5-km run test. Although miR-21 level was increased, there was no statistical difference between the two groups. Notably, our data were not completely consistent with previous exercise-related studies. Mooren et al. (2014) reported that miR-21 and miR-155 were not affected by marathon run, while miR-1 expression increased in the health training population, demonstrating the potential role of miR-1 in cardiovascular adaptation processes after endurance exercise. Ramos et al. (2018) found that the expressions of miR-21 and miR-210 were non-responsive to exercise, while miR-146a expression was responsive to exercise at some threshold but without dose dependence. Furthermore, miR-1 level is responsive to exercise with dose dependence to increasing intensity (Ramos et al., 2018). Nevertheless, Cui et al. (2017) reported that miR-21and miR-181a levels significantly increased after exercise, but miR-1 level remained unchanged. Combined with the results of previous and this study, we speculate that the alternation of specific miRNAs during exercise is related to the type, intensity, duration, and frequency of exercise and subject status and monitoring time point of miRNAs after exercise. this study, we provided new sights in miRNAs expression profiles during the 5-km run test.

Serum cTNI, Myo, CK-MB, AST, and LDH are classic markers of myocardial injury. They are closely related to acute cardiovascular events, such as acute coronary syndrome and can be used as reference indicators for predicting cardiovascular events. Previous studies have documented that during exercise, the metabolic rate of the body speeds up and oxygen demand increases dramatically, but the pumping of blood by the heart and blood oxygen supply fail to increase correspondingly, which may cause acute coronary ischemia and hypoxia (Schwartz and Corrado, 2012). Xu et al. (2016) reported that an acute exercise increased the levels of CK, LDH, and N-terminal fragment of the BNP precursor (NT-proBNP), but did not affected CK-MB, cardiac troponin T (cTNT), and high-sensitivity C-reactive protein (hs-CRP) in subjects. However, their study was conducted in patients with congestive heart failure. In this study, we found that serum Myo, CK-MB, AST, LDH, and IL-6 were significantly increased, while the levels of IMA were significantly decreased after the 5-km running training, indicating that a distinct exercise adaptation in young healthy subjects. Additionally, it is demonstrated that exercise can enhance the release of IL-6 in muscles and induce anti-inflammatory responses in the body by promoting IL-10 secretion and inhibiting IL-1 release (Sharif et al., 2018). Although most studies suggest that IMA can be significantly increased in severe myocardial ischemia, such as coronary heart disease (Lippi et al., 2005), it may show a reduced efficacy in identifying myocardial ischemia in exercise-induced cardiac or skeletal muscle ischemia (Apple et al., 2005), owing to interference by a number of factors, such as the presence of circulating lactate and the increased total albumin inherent to prolonged exercise. These data suggest that the physical state of participants, exercise type, intensity, and duration affect levels of these biomarkers.

Further correlation analysis demonstrated that the five altered miRNAs were correlated with serum indicators. This study showed that miR-1, miR-146a, miR-181, and miR-155 were positively related to IL-6 and all the five miRNAs were negatively correlated with IMA. Xie et al. (2019) have reported that miR-146a inhibits inflammatory response and myocardial injury through negative feedback of the toll-like receptor 4 (TLR4)/transcription factor nuclear factor (NF)-κB signaling pathway, whereas miR-1, miR-181, and miR-155 were shown to suppress the degree of inflammatory response in myocardial hypoxia state by regulating their target genes (Schuttler et al., 2019; Xie et al., 2019). Furthermore, most of the published studies have proven that IAM is a pivotal inflammatory marker for multiple diseases, such as rheumatoid arthritis, transient ischemic attack, vascular disease, and so on (Shaker et al., 2019; Balta, 2021; Li et al., 2021). It seems reasonable to hypothesize that miRNAs may suppress the inflammatory response to decrease IAM levels during exercise training by regulating IL-6 release. Meanwhile, in this study, miR-146a, miR-155, and miR-210 showed a positive correlation with Myo, CK-MB, and LDH, respectively. To the best of our knowledge, this study showed for the first time to identify the relationship among miRNAs and Myo, CK-MB, LDH, and IMA in exercise. Those results indicate that altered miRNAs might affect the levels of inflammation and myocardial injury markers by regulating the inflammatory status of young subjects. However, whether these miRNAs directly regulate the above biochemical parameters still needs further exploration. Furthermore, the logistic regression analysis results showed that miR-146a, AST, LDH, and IL-6 are potential risk factors and IMA is a potential protective factor in exercise adaptation. This reminds us that when subjects have long-term excessive military training, increased expression of miRNAs may impair cardiovascular function, leading to increased myocardial cell damage and causing significant changes in myocardial enzyme spectrum, inflammatory factors, and a series of indicators. The identification of specific miRNAs has opened a new field of mechanism modulating clinical biochemical parameters of cardiovascular adaptation during the 5-km run test in subjects.

Nevertheless, several limitations of this study should be acknowledged. One of the weaknesses is the relatively small size, which may be due to special restrictions of military institutions. Another notable issue is that how miRNAs regulate those biochemical parameters during exercise is unclear. Therefore, further large-scale studies are required to clarify the underlying mechanism of miRNAs participation in cardiovascular adaptation during exercise.

## Conclusion

We identified miR-1, miR-146a, miR-155, miR-181, and miR-210 elevated during exercise training; these miRNAs show significantly correlation with IL-6, IMA, Myo, CK-MB, and LDH. Additionally, miR-146a, AST, LDH, and IL-6 showed potential risk factors and IMA is a potential protective factor in exercise adaptation. These results suggest that miRNAs might play an important role as a physiological mediator in the cardiovascular function adaptation of exercise, which provides new insights and directions for understanding the cardiovascular system adaptation status of subjects in training and carrying out scientific and reasonable training in the future.

## Data Availability Statement

The original contributions presented in the study are included in the article/supplementary material, further inquiries can be directed to the corresponding authors.

## Ethics Statement

The studies involving human participants were approved by the Research Ethics Committee of Jinling Hospital (2018NZGKJ-096). The patients/participants provided their written informed consent to participate in this study.

## Author Contributions

JW, CW, and DL participated and conceived the study design. DL, PW, and WW collected the data. DL, PW, and CW performed the experiments and analyzed the data. YZ, LL, and CW interpreted and discussed the data. DL wrote the manuscript. CW and JW refined the final draft and revised the manuscript. All the authors reviewed the final version of the manuscript.

## Conflict of Interest

The authors declare that the research was conducted in the absence of any commercial or financial relationships that could be construed as a potential conflict of interest.

## Publisher’s Note

All claims expressed in this article are solely those of the authors and do not necessarily represent those of their affiliated organizations, or those of the publisher, the editors and the reviewers. Any product that may be evaluated in this article, or claim that may be made by its manufacturer, is not guaranteed or endorsed by the publisher.
